# ACEMg Diet Supplement Modifies Progression of Hereditary Deafness

**DOI:** 10.1038/srep22690

**Published:** 2016-03-11

**Authors:** Kari L. Green, Donald L. Swiderski, Diane M. Prieskorn, Susan J. DeRemer, Lisa A. Beyer, Josef M. Miller, Glenn E. Green, Yehoash Raphael

**Affiliations:** 1 Kresge Hearing Research Institute, Department of Otolaryngology - Head and Neck Surgery, The University of Michigan, Ann Arbor, MI, USA

## Abstract

Dietary supplements consisting of beta-carotene (precursor to vitamin A), vitamins C and E and the mineral magnesium (ACEMg) can be beneficial for reducing hearing loss due to aminoglycosides and overstimulation. This regimen also slowed progression of deafness for a boy with GJB2 (CONNEXIN 26) mutations. To assess the potential for treating GJB2 and other forms of hereditary hearing loss with ACEMg, we tested the influence of ACEMg on the cochlea and hearing of mouse models for two human mutations: GJB2, the leading cause of childhood deafness, and DIAPH3, a cause of auditory neuropathy. One group of mice modeling GJB2 (Gjb2-CKO) received ACEMg diet starting shortly after they were weaned (4 weeks) until 16 weeks of age. Another group of Gjb2-CKO mice received ACEMg in utero and after weaning. The ACEMg diet was given to mice modeling DIAPH3 (Diap3-Tg) after weaning (4 weeks) until 12 weeks of age. Control groups received food pellets without the ACEMg supplement. Hearing thresholds measured by auditory brainstem response were significantly better for Gjb2-CKO mice fed ACEMg than for the control diet group. In contrast, Diap3-Tg mice displayed worse thresholds than controls. These results indicate that ACEMg supplementation can influence the progression of genetic hearing loss.

Congenital sensorineural hearing loss (SNHL) is the most common sensory deficit in the US, affecting at least 0.1% of all children^1^. In developed countries, genetic mutations account for more than half of all cases of congenital SNHL. Not all neonates with SNHL are profoundly deaf and some mutations may not lead to measurable hearing loss until later in life^2^,^3^,^4^. For these cases, it is important to identify therapies that prevent progression and reverse loss of functionality. Gene therapy approaches aimed at these goals are under investigation in many labs^5^,^6^,^7^,^8^,^9^ but are not yet ready for clinical use. In this study, we examined the potential of an alternative strategy of dietary supplementation to enhance the overall homeostatic health of cells in the cochlea in order to improve their function and slow or prevent their pathology.

A common factor in hearing loss caused by overstimulation or ototoxicity is the generation of free radicals and oxidative stress^10^,^11^,^12^,^13^,^14^,^15^. Antioxidants have been shown to reduce the impact of oxidative stress in neuronal disorders^16^,^17^,^18^,^19^, cancer^20^,^21^,^22^, heart disease^23^,^24^, inflammatory diseases^25^,^26^,^27^, and ischemia-reperfusion injuries^28^,^29^. In the inner ear, reduction of oxidative stress related to overstimulation or aminoglycoside ototoxicity has been demonstrated to protect the sensory hair cells and hearing^12^,^30^,^31^. Treatment with antioxidants also has been shown to preserve gap junction function^32^,^33^, which is especially relevant because mutations in *GJB2*, which encodes the gap junction protein CONNEXIN 26 (Cx26), account for over half of all hereditary deafness cases^34^. With dysfunctional gap junctions in the cochlea, it would be especially important to remove the excessive potassium ions, thereby protecting mitochondria from damage that can be caused by this potent free radical^35^.

Additional motivation for investigating the efficacy of antioxidant supplements in remediation of genetic hearing loss was the positive effect of a diet augmented with antioxidants (vitamin A precursor, C and E) and a vasodilator (magnesium), hereafter ACEMg, on a human patient with progressive Cx26 hearing loss^36^. Administration of this regimen over a three-year period not only stopped the progression of hearing loss, but also resulted in a small hearing improvement. In light of these results, we assessed the influence of this potentially protective ACEMg dietary supplement on the progression of hearing loss in mouse models for two different forms of human hereditary deafness: Cx26 and AUNA1.

The *Gjb2*-CKO model was chosen because of the high frequency of mutations of the gene encoding this protein. The carrier rate of *GJB2* mutations known to cause Cx26 related deafness is greater than 3% of all individuals in the United States and is among the highest of any disease^37^. Many individuals with *GJB2* mutations are not profoundly deaf and their hearing is typically stable^38^. However, in other cases, the hearing loss is relentlessly progressive^39^,^40^. Moreover, some profoundly deaf children, with biallelic *GJB2* mutations, pass their newborn hearing screening, indicative of near normal hearing at birth, and then have subsequent degradation of hearing^41^,^42^. These patterns suggest that cells present at birth may be amenable to rescue.

The AUNA1 model (*Diap3*-Tg) was chosen because it is progressive, indicating there are cells present to be rescued, but the role of the protein and the consequences of the mutation are very different in nature from the connexin mutation. AUNA1 is a very rare form of progressive hearing loss with late onset in humans, typically developing in the second decade of life^2^,^3^,^4^. Clinical manifestation of AUNA1 is typical of auditory neuropathy where outer hair cell function is initially preserved but the function of the auditory nerve is impaired. As the hearing loss progresses, outer hair cell function also is affected^4^. The progression is rapid, and profound deafness typically results within 20 years^3^,^4^.

We provided the ACEMg enhanced diet to mice with a conditional deletion of *Gjb2* (*Gjb2*-CKO) beginning at 4 weeks, one week after weaning. In a second group of *Gjb2*-CKO mice, the enhanced diet was provide to mothers beginning 1 week prior to mating and the offspring were weaned onto that diet. The *Gjb2*-CKO mice given the enhanced diet after weaning had profound hearing loss before initiating the enhanced diet, but their hearing loss did not progress and a small yet significant improvement was seen. The *Gjb2*-CKO mice whose mothers received the enhanced diet during gestation had slightly better hearing at 4 weeks than offspring of mothers fed the standard diet and their hearing loss neither worsened nor improved subsequently. Hearing loss in control animals continued to demonstrate deterioration. In contrast, the hearing loss of *Diap3*-Tg mice given the ACEMg beginning at 4 weeks of age progressed much more rapidly compared to *Diap3*-Tg mice given control diet. These results suggest that dietary supplementation can influence the inner ear phenotype caused by mutations and that the influence can be negative or positive depending on the mutation.

## Results

### General Health

The mice given the ACEMg diet did not have any apparent negative health defects and gained weight appropriately. Pregnant breeder mice demonstrated no apparent adverse effects from the ACEMg diet. Mouse litters consisted of both homozygous and heterozygous pups at the expected 1:1 ratio (38/78 mouse pups were homozygotes). Pups were born without any readily apparent health defects. The mice were small but gained weight well. The ACEMg diet did not negatively affect litter size, pup weight, or health. The feces of the mice given the ACEMg diet were orange in color due to the vitamins, but they were normal in consistency.

Mice in the prenatal ACEMg diet group had higher mortality under anesthesia than did control mice. Of the first 16 mice in the prenatal ACEMg diet group, 7 died under anesthesia given for ABR testing. No control diet mice died under anesthesia. The increased anesthesia-related mortality may have been due to peripheral vasodilation caused by the anesthetic regimen combined with the diuretic effect from vitamin C and vasodilation induced by magnesium. The increased anesthetic mortality rate was resolved by giving subcutaneous fluids prior to ABR testing and yohimbine (7 mg/kg) after completion of ABR testing. This modified protocol was used with all subsequent mice in all diet groups and no additional mice died under anesthesia.

### ABR Data

The 4 week thresholds of *Gjb2*-CKO mice in all groups were variable with a standard deviation of approximately 10 dB at each frequency. All *Gjb2-*CKO mice had substantial hearing loss at this early age. In some cases, thresholds could not be detected even at the maximum stimulation level.

### *Gjb2*-CKO Mice, Post-Weaning Intervention

**Figure 1:**
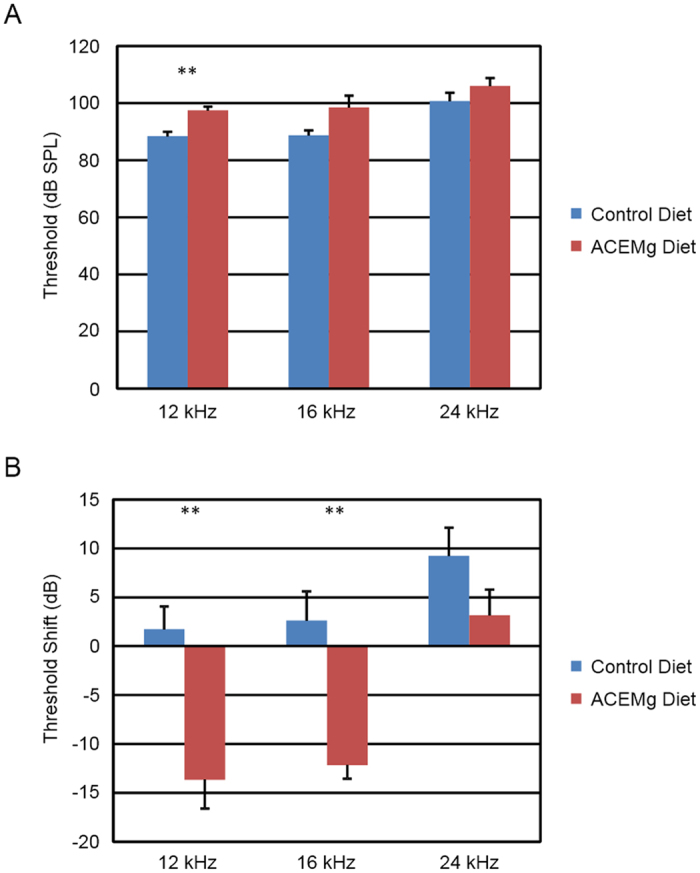
Post-weaning dietary supplement slowed or reversed hearing loss in *Gjb2*-CKO mutant mice. (**A**) ABR thresholds at 4 weeks of age demonstrate substantial hearing loss prior to supplementation, and a small pre-treatment difference between groups assigned to the two diets, which was significant only at 12 kHz. (**B**) Changes in ABR thresholds between 4 and 16 weeks of age were significantly different between the diet groups, with ACEMg diet group having significant improvement at 12 kHz and significantly less additional loss at 24 kHz. Negative shifts indicate improved hearing. Bars indicate mean +/− standard error. **indicates p < 0.01.

Because allocation of mice to experimental groups was blind to their 4 week hearing threshold, and was conducted over several weeks, statistical analysis of differences between mice assigned to those groups was not performed until all mice had been assigned to a group. That statistical analysis revealed a significant difference in ABR thresholds between the mice that were assigned to the two diet groups (F_3,10_ = 5.86, p = 0.014). Post hoc analyses showed that animals switched to the enhanced diet had higher average thresholds at 12 kHz than animals switched to the control diet; significant differences were not found at 16 kHz or 24 kHz (Fig. 1A).

At 16 weeks of age (after 12 weeks on the diets), the change over 12 weeks differed significantly between diets (F_3,10_ = 9.76, p = 0.0026). On average, thresholds of animals on the ACEMg diet decreased (i.e. improved) more than 10 dB at 12 and 16 kHz, whereas average thresholds of animals on the control diet increased (i.e. worsened) at least 2 dB at those same frequencies (Fig. 1B). These differences were statistically significant by sequential Bonferroni criteria. At 24 kHz, both groups had positive threshold shifts that were not significantly different.

### *Gjb2*-CKO Mice, Prenatal Intervention

**Figure 2:**
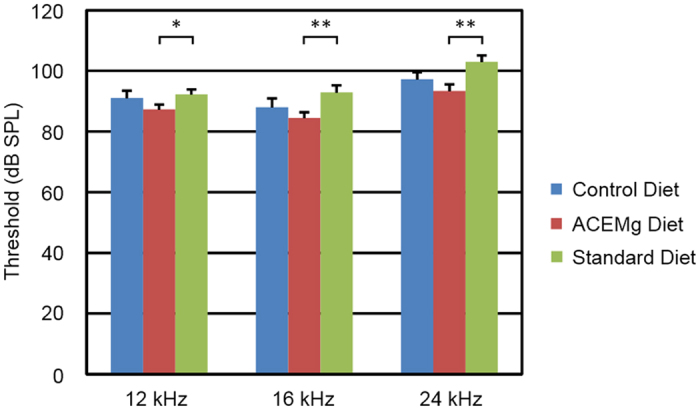
*Gjb2*-CKO mutant offspring of dams fed ACEMg diet had lower thresholds at 4 weeks of age than mutant offspring of dams fed control or standard diets. ANOVA indicates a significant effect of diet, but post-hoc comparison found significant differences only between the ACEMg and standard diet groups. Bars indicate mean +/− standard error. Brackets indicate significant pairwise comparisons: *p < 0.05, **p < 0.01.

We performed a 3-way analysis comparing hearing at 4 weeks of age in the two prenatal groups (control and enhanced diet) and all animals on standard diet until 4 weeks (before they were switched to control or enhanced diets) and did not find a significant effect of diet in this 3-way test (F_6,80_ = 1.72, p = 0.13). The two most different groups were the prenatal ACEMg group and the standard diet group (Fig. 2). Post hoc pairwise tests, using the sequential Bonferroni criterion for significance showed that there was a difference between the prenatal ACEMg group and the standard diet group (F_3,27_ = 3.72, p = 0.023), but not between prenatal ACEMg and prenatal control groups, or between prenatal control and the standard diet groups.

**Figure 3:**
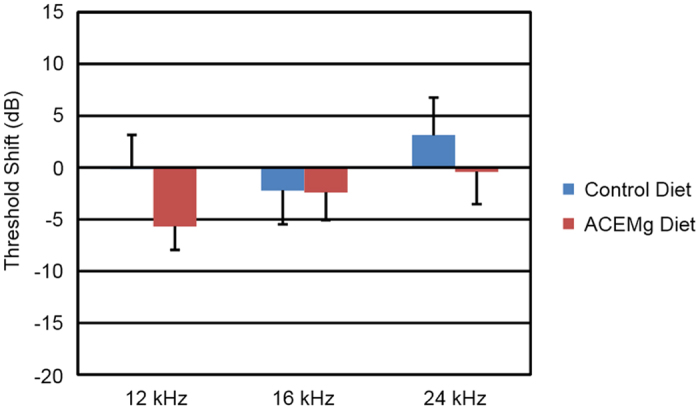
*Gjb2*-CKO mutant mice maintained on the maternal prenatal diet did not have significantly different changes in hearing between 4 and 12 weeks of age. Bars indicate mean +/− standard error.

**Figure 4:**
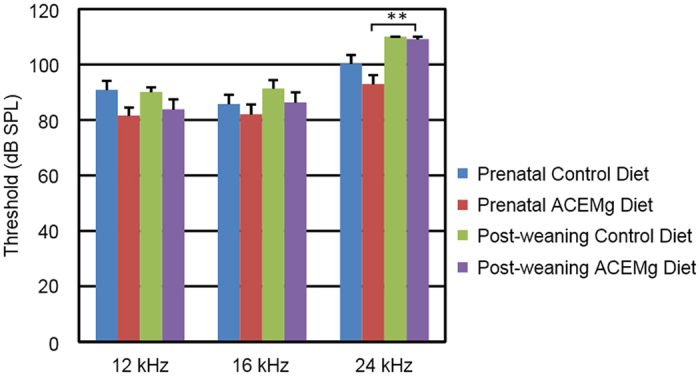
ABR thresholds at ending time points for *Gjb2*-CKO mutant mice in all four diet intervention groups. At all frequencies, the lowest means are seen in the prenatal ACEMg group, but significant differences are seen only at 24 kHz, between this group and both post-weaning groups. Bars indicate mean +/− standard error. **indicates p < 0.01.

In both prenatal groups, threshold shifts between 4 and 12 weeks were small and not significantly different (F_3,26_ = 1.56, p = 0.22). In fact, neither group had appreciable change over this interval (Fig. 3). The animals in both prenatal intervention groups tended to have lower thresholds at 12 weeks of age than animals switched onto those diets from standard chow for 12 weeks (Fig. 4). At their ending time points, the prenatal ACEMg group had the lowest (i.e. best) average thresholds at all three frequencies. The difference between prenatal ACEMg and prenatal control diet groups was significant (F_3,26_ = 3.42, p = 0.032). Thresholds of the prenatal control group were not different from either post-weaning group. Differences between the prenatal ACEMg group and both post-weaning groups were significant (F_9,120_ = 2.92, p = 0.0036), mainly due to large differences at 24 kHz (prenatal ACEMg vs post-weaning ACEMg p = 0.0096; prenatal ACEMg vs post-weaning control p = 0.00206).

### Diap3-Tg Mice, Post-Weaning Intervention

**Figure 5:**
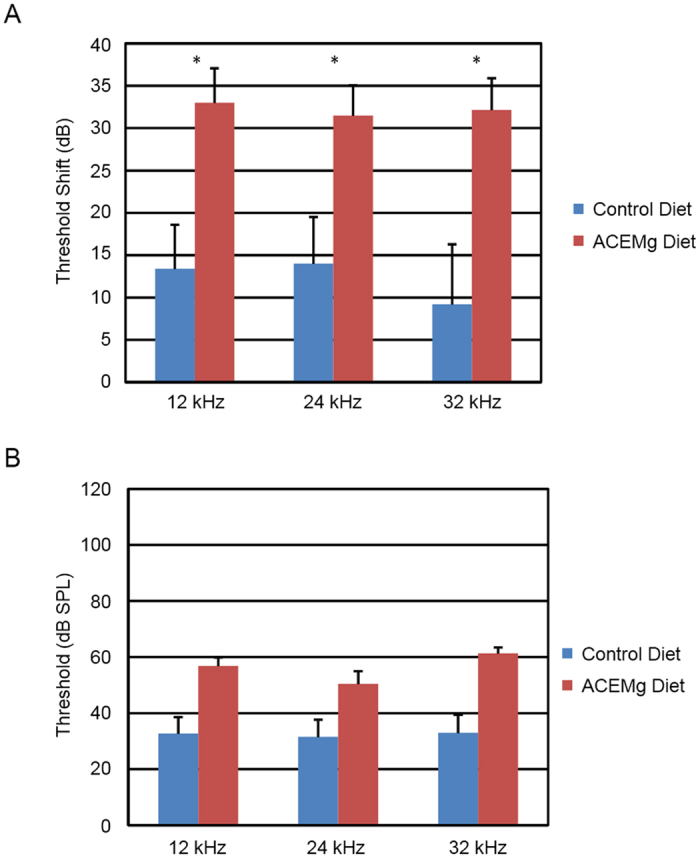
Post-weaning dietary supplement slowed or reversed hearing loss in *Diap3*-Tg mutant mice. (**A**) ABR threshold shifts between 4 and 12 weeks in mutant mice on the diet were twice the shifts of mutant mice on the control diet, and significantly different at all frequencies. (**B**) The resulting endpoint thresholds were higher, indicating worse hearing, in Diap3-Tg mutant mice on the ACEMg diet. Bars indicate mean +/− standard error. *indicates p < 0.05.

At 4 weeks of age, prior to the start of treatment with the diet, mice underwent ABR testing. Initial thresholds of *Diap3*-Tg mice were highly variable, means of mice allocated to the control or ACEMg diets differed by 4 dB, which was not significant (F_3,7_ = 0.81, p = 0.53). After 8 weeks on the diet (12 weeks of age), both diet groups exhibited large threshold shifts compared to the 4 week time point, but the threshold shifts were significantly higher (i.e. worse) in mice given the ACEMg diet than in mice given the control diet (F_3,7_ = 4.55, p = 0.045). Mice given the enhanced diet had an average shift of 32 dB across frequencies, more than twice that experienced by mice on the control diet (Fig. 5A), resulting in a more severe hearing loss (Fig. 5B).

### Histology

#### Post-weaning Intervention Gjb2-CKO Mice

**Figure 6:**
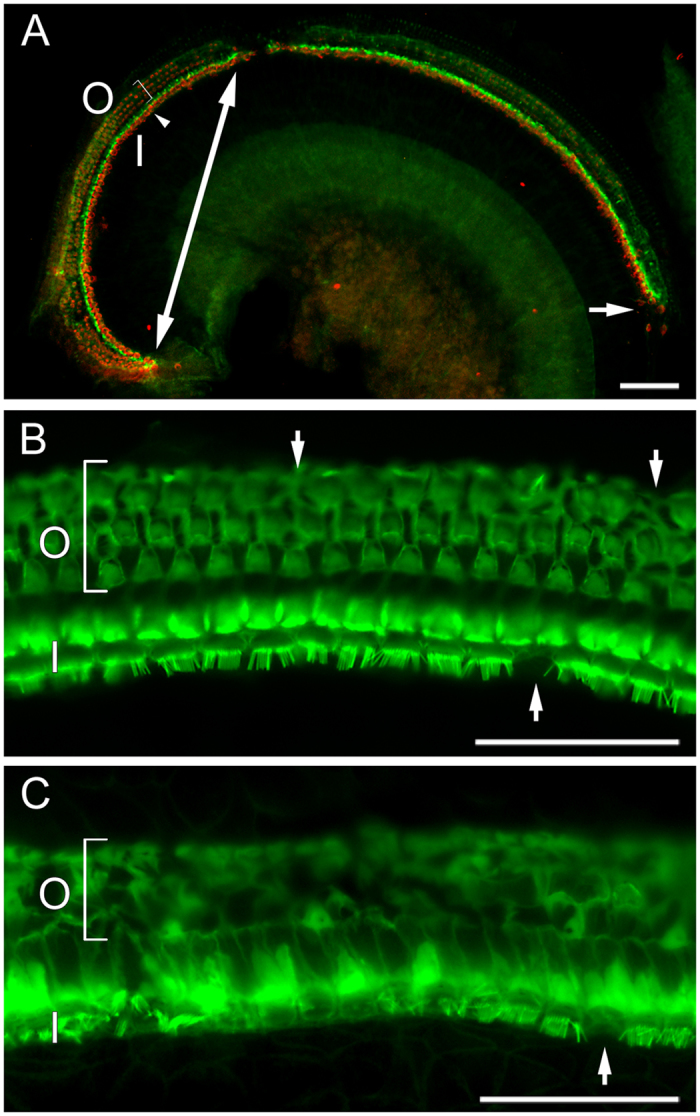
Epifluorescence images of auditory epithelium from a *Gjb2*-CKO mouse on the control diet showing survival of outer hair cells is restricted to an area close to the apex. (**A**) Low magnification image labeled to show actin (green) and myosin7a (red). In the most apical region, between tips of the double-headed arrow, ordered rows of inner hair cells (I, arrowhead) and outer hair cells (O, bracket) can be seen. More basally, are regions with only inner hair cells (to single-headed arrow), and no hair cells (below arrow). Bar = 100 μm. (**B**) Higher magnification images near the apex with actin labelling, showing surviving hair cells in well-ordered rows; locations of missing cells (arrows) are easily recognized as gaps in this array. (O and I indicate outer and inner hair cells as above). Bar = 50 μm. (**C**) Higher magnification images of region where only inner hair cells survive. Note disorganized appearance of the region where outer hair cells normally would be found. Bar = 50 μm.

Epifluorescence examination of phalloidin-labeled whole mounts of the organ of Corti revealed substantial loss of hair cells in both the mice on the control and the ACEMg diet (Fig. 6). In the basal turn of the cochlea >2.47 mm from the apex, no hair cells were seen in mice in either diet group. At <2.47 mm from the apex, there was some hair cell preservation, but it was not significantly different between diet groups for inner or outer hair cells (inners p = 0.66, outers p = 0.57).

#### Prenatal Intervention Gjb2-CKO Mice

**Figure 7:**
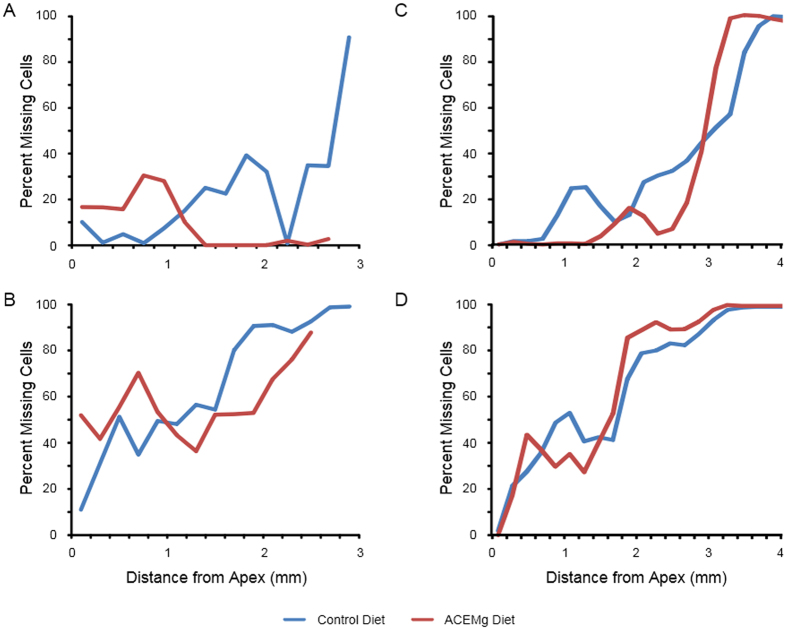
Average loss of inner hair cells (**A,C**) and outer hair cells (**B,D**) as a function of distance from the cochlear apex for *Gjb2*-CKO mice in post-weaning (**A,B**) and pre-natal (**C,D**) intervention groups. Regions farther from the apex than those shown averaged 100% hair cell loss. Significant reduction of hair cell loss was seen only in inner hair cells of the preweaning intervention group (**C**), and then only in the region <3.04 mm from the apex.

Mice raised on either diet also had nearly complete inner hair cell loss >3.42 mm from the apex (Fig. 7). Between 3.04 and 3.42 mm, difference in inner hair cell survival was not significant, but in the larger region <3.04 mm, mice raised on the ACEMg diet did have greater inner hair cell preservation (p = 0.043). Greater hair cell survival in this region is consistent with the ABR results showing these mice had better hearing at the lowest frequency tested (12 kHz), which is the one transduced in the region closest to the apex. There was no significant difference in outer hair cell preservation between mice on the ACEMg diet and the mice on the control diet in any region of the cochlea.

#### Diap3-Tg Mice

**Figure 8:**
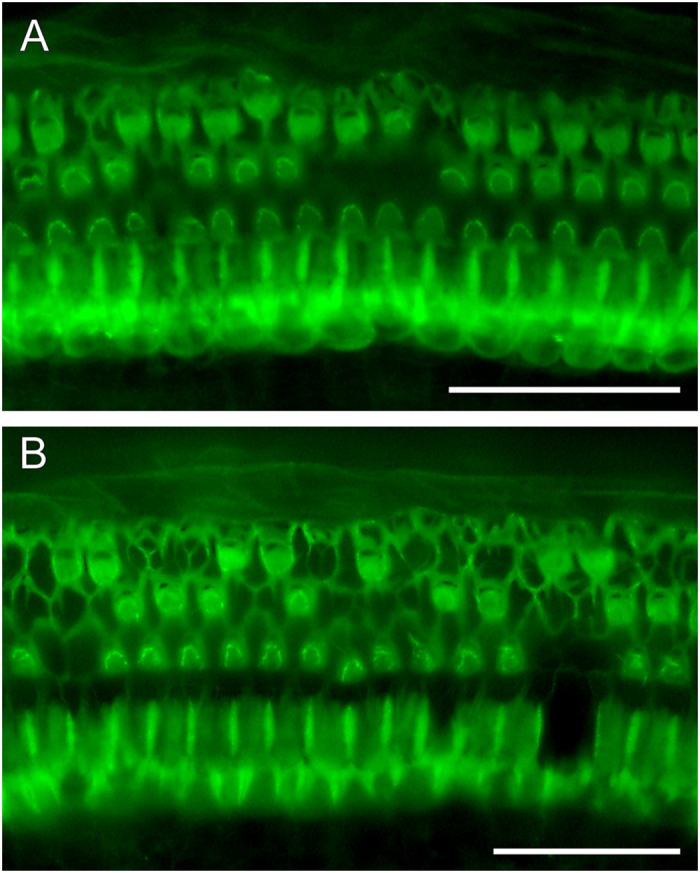
Epifluorescence images of comparable mid-apical regions of auditory epithelium from *Diap3*-Tg mice on the control diet (**A**) and ACEMg diet (**B**). Bar = 50 μm.

Hair cells of Diap3-Tg animals were not counted, but qualitative images were obtained from 6 mice; 2 given the ACEMg diet and 4 given the control diet (Fig. 8). There was a notable difference in the quality of the organ of Corti in the mid apex between the mice given the control diet (Fig. 8A) and the mice given the ACEMg diet (Fig. 8B) with mice on the control diet retaining a higher proportion of hair cells.

## Discussion

A diet supplemented with ACEMg was given to two mouse models for human hereditary deafness (*GJB2* and AUNA1) to test whether the diet would influence the progression of hearing loss and associated inner ear pathology. In the *Gjb2*-CKO mice, the ACEMg diet reduced the progression of hearing loss and elicited a small, but significant improvement in hearing thresholds. This functional improvement was associated with mild but significant enhancement of hair cell preservation. In contrast, the diet had a deleterious effect in the *Diap3*-Tg mouse (AUNA1), exacerbating hearing loss in comparison to *Diap3*-Tg mice given the control diet.

Absence of gap junctions containing Cx26, due to *GJB2* mutations, may lead to chemical imbalances in extracellular space surrounding hair cells, and ultimately to hair cell death and hearing loss. Mutations in *GJB2* decrease effective shuttling of cellular by-products, including K^+^ and other free radicals, by the supporting cells^43^. Increased oxidative stress may result from the local extracellular accumulation of potassium ions and reactive oxygen species (ROS) associated with the absence of Cx26^44^. Several different potassium ion channels are present in mitochondrial membranes^45^,^46^ and contribute to mitochondrial function^47^ and their role in the demise of cells when potassium levels are excessive^48^. The stress induced by the excessive potassium may also increase phagocytic activity of Deiters cells, reported to be part of the process of eliminating hair cells^49^,^50^. The improvement seen in these mice is consistent with the hypothesis that antioxidant supplements should reduce the oxidative stress and hair cell loss. Previous studies demonstrated that systemically administered ACEMg reduces intense noise- and aminoglycoside-induced free radical formation in the supporting cells and hair cells^51^,^52^. Here we demonstrate that ACEMg may be effective in reducing cell death and hearing impairment associated with the hereditary deafness induced by Cx26 mutations.

In contrast to the positive results seen in the *Gjb2*-CKO mice, the ACEMg diet had a detrimental effect on mice with AUNA1 hearing loss. These mice exhibited more severe progression of hearing loss than mice given the control diet. AUNA1 mutation leads to overexpression of *Diap3* (human *DIAPH3*), a ubiquitously expressed member of a group of proteins that are important in regulation of actin networks^53^. In the ear, overexpression of the protein causes profound abnormalities in the stereocilia of the IHC and loss of IHC-auditory nerve synapses^3^. Outer hair cell function is preserved, but IHC and auditory nerve function is disrupted. We hypothesize that the detrimental effects of the ACEMg supplement in the inner ears of *Diap3*-Tg mice reflect amplification of the overexpression that produces the abnormalities in the IHC and/or auditory nerve.

Specifically tailored nutritional supplementation has been shown to be of benefit for some forms of deafness when it addresses the underlying metabolic abnormality. For instance, hearing loss and cretinism that are associated with iodine deficiency can be readily prevented by dietary supplementation^54^. However, supplements may have unintended negative effects. In our study, antioxidant supplements improved hearing in a mutation expected to increase oxidative stress, but worsened hearing in a mutation that overexpresses a protein. When the specific function of *Diaph3* is elucidated, it may shed light on the reasons for the negative influence of the ACEMg diet on the hearing of these mutant mice. The detrimental outcome of the ACEMg diet on the hearing of *Diap3*-Tg mutant animals alerts to the fact that diet supplementation can have a negative impact and highlights the need to test outcomes of supplementation and tailor supplement regimens for specific mutations.

Our *Gjb2*-CKO mice exhibit severe hearing loss soon after weaning. The deafness progresses with age and is accompanied by progressive hair cell loss and auditory nerve degeneration^55^. Thus, these mice exhibit a phenotype that is much more severe than most human cases of Cx26-related hearing loss. However, our mouse model may more closely resemble the subset of human cases that does have severe deafness at birth and subsequent progression. In a child with a more severe, rapidly progressing Cx26 hearing loss, treatment with an ACEMg regimen over a period of 3 years halted the progression and produced a slight improvement in hearing^36^. The small improvements seen in this case, and in our mice, can have a substantial impact on functionality, quality of life, educational opportunities, income, and ability to integrate into society^56^,^57^. The significant benefits demonstrated hold out the promise that antioxidants may be effective for treating progressive Cx26-related hearing loss in children. The lack of side effects provides strong incentive to further investigate the mechanism and the clinical utility of providing ACEMg to *GJB2* patients.

In conclusion, ACEMg significantly influenced deafness progression in the cochleae of both *Gjb2*-CKO and *Diap3*-mice. In *Diap3*-Tg mice, there was a deleterious effect whereas in *Gjb2*-CKO mice there was significant improvement of auditory thresholds. These data link dietary supplements with modulation of phenotypes in hereditary deafness and suggest that effects may vary depending on the mutation. The positive influence of ACEMg on structure and function in *Gjb2*-CKO ears is especially important considering the prevalence of Cx26 deafness.

## Methods

### Animals

Animal care, handling and all procedures described in this work were performed using accepted veterinary standards and were approved by the University Committee on the Use and Care of Animals of the University of Michigan.

The *Gjb2*-CKO mouse model is a conditional knockout developed by crossing Cx26^loxp/loxp^ mice with two lox sites around exon 2 of the *GJB2* gene^44^ and *Sox10*Cre mice^58^. In the cochlear epithelium area, Sox10 is expressed in the non-sensory cells around the organ of Corti^59^. The two lines were outcrossed to develop wild type mice then the wild type mice were crossed in order to achieve a double transgenic mouse (Cx26^Sox10Cre^), as previously described^55^. The heterozygous mutant mice did not have significantly different thresholds from the wild type mice, however, the homozygous mutant mice exhibit profound, progressive hearing loss, representative of the worst-case hearing impairment in humans with *GJB2* mutations. Only the homozygous mice were used in this study on the ability of the ACEMg diet to prevent hearing loss in this *Gjb2*-CKO mouse.

The AUNA1 mouse model, *Diap3*-Tg, developed by Schoen *et al.*^3^, is a transgenic mouse in which overexpression of *Diap3* (the ortholog of human *DIAPH3*) is driven by the immediate-early enhancer of the HCMV (human cytomegalovirus) with chicken BETA-ACTIN/rabbit BETA-GLOBIN hybrid (CAG) promoter. FVB/NJ mice were mated with the surviving founders and two lines of offspring with the transgene exhibited hearing loss within 16 weeks of age. In these mice, *Diap3* overexpression causes progressively severe abnormalities in the stereocilia of the inner hair cells and loss of inner hair cell synapses with the auditory nerve. Line FVB-Tg(CAG-*Diap3*)924Lesp/J was selected for use in this study because the mice presented with severe hearing loss (35 dB) at 16 weeks of age, but had no significant hearing loss at 4 or 8 weeks of age.

In all groups, litters were weaned at 3 weeks of age and genotyped. Genomic DNA was prepared from the pups by incubation of ear biopsies in DNA extraction buffer. DNA was amplified by PCR under standard conditions. For *Gjb2*-CKO mice, the homozygous pups were identified and used in the study; heterozygous mouse pups were excluded. All *Diap3*-Tg mice with the transgene were identified and used in the study.

### Diet Supplement and Regimen

An experimental diet enhanced with vitamin A precursor beta-carotene, vitamins C, E, and magnesium (ACEMg) (Harlan Teklad Diet TD.110032) and a control diet (Harlan Teklad Diet TD.110031) were developed. The ACEMg diet was a tocopherol-stripped soy-based diet supplemented with 1.05 g/kg consumer supplied beta-carotene (Kingchem, Allendale, NJ), 10.29 g/kg vitamin C, 7.76 g/kg vitamin E, and 13.48 g/kg magnesium. The control diet was the same tocopherol-stripped soy-based diet without the added A, C, E, and magnesium. Before the onset of the feeding with the experimental diets, animals were on a standard diet (PicoLab Diet 5L0D) prepared from ground corn, dehulled soybean meal, beet pulp, fishmeal, and other plant and animal materials. Fresh diet was provided daily to the mice.

Two protocols of diet supplementation were used. In one protocol, used for all *Diap3*-Tg mice and one group of *Gjb2*-CKO mice, individuals were transitioned from standard chow to the ACEMg or designed control at 4 weeks, 1 week after weaning. In the other protocol, used only for *Gjb2*-CKO mice, mothers were fed the ACEMg or control diet from 1 week before they were placed in a cage with a male. They continued to be fed the ACEMg throughout gestation and lactation and offspring were then weaned onto that same diet. The purpose of the prenatal protocol was to test the hypothesis that earlier intervention time point may be more effective at preventing the progression in hearing loss associated with the Cx26 mutation. All diets were provided ad libitum, and all mice were maintained on the designated diet until the end of the experiment. Numbers of individuals in each group are given in Table 1.

### Auditory Brainstem Response Measurement

Hearing in all included mice was tested with standard techniques by measuring thresholds for auditory brainstem responses (ABR) to tonal stimuli. ABRs were measured in the left ear of each mouse. *Gjb2*-CKO mice in the post-weaning intervention groups were evaluated 4 and 16 weeks of age; *Gjb2*-CKO mice in the prenatal intervention group were evaluated at 4 and 12 weeks of age. In both groups, ABRs were measured in the left ear at 12, 16, and 24 kHz. The *Diap3*-Tg mice were evaluated at 4 and 12 weeks of age in the left ear at 12, 24 and 32 kHz.

Prior to testing, a standard intraperitoneal anesthetic regimen of ketamine (65 mg/kg) (Hospira Inc., Lake Forest, IL), xylazine (7 mg/kg) (Lloyd Laboratories, Shenandoah, IA) and acepromazine (2 mg/kg) (Boehringer Ingelheim Vetmedica, St. Joseph, MO) was administered. Subcutaneous glycopyrrolate (0.2 mg/kg) (West-Ward Pharmaceuticals, Eatontown, NJ) was administered to stabilize heart rate and decrease the production of saliva while the mice are anesthetized. To maintain body temperature while anesthetized, mice were placed on a water-circulating heating pad. To maintain anesthesia depth, additional ketamine was administered as needed. To assist with recovery from anesthesia, yohimbine was administered (7 mg/kg, S.C.) (Lloyd Laboratories, Shenandoah, IA).

An operating microscope was used to ensure the outer ear canal was free of wax, and that the canal, tympanic membrane, and visible portions of the malleus were free from deformities. Needle electrodes were then placed subcutaneously at the vertex (active), and inferior to the pinnae of the test ear (reference) and the contralateral ear (ground).

All ABR measurements were performed in an electrically and acoustically shielded chamber (C A Tegner AB, Bromma, Sweden). Acoustic stimuli were pure tone bursts with a 15 ms duration and 1 ms rise and fall times, at 10 bursts per second. Acoustic stimuli were generated using Tucker–Davis Technology (TDT, Alachua, FL) System III hardware and SigGen/BioSig software. A tube connected to a transducer (Beyer B4-31.05-00 headphone element; Beyer Dynamic, Farmingdale, NY) was used to present the stimuli to the external auditory meatus. Starting levels were 90–105 dB and sound intensity was decreased until threshold was reached. The smallest step size was 5 dB. Neural responses from up to 1024 tone bursts were amplified (100,000×), filtered (300–3000 Hz) and averaged using SigGen/BioSig software.

The threshold was defined as the lowest intensity of stimulation that produced a repeatable response based on an identifiable ABR waveform. The software system was recalibrated in between the two groups of *Gjb2*-CKO mice. The maximum stimulation level was 110 dB for the post-weaning intervention group and 105 dB for the prenatal intervention group. To correct for this difference, mice with no measurable responses at a frequency, or with a measurable response greater than 105 dB, were scored as having a response at 110 dB, the next possible level above 105 dB.

### Hair Cell Assessment

After the final ABR, the deeply anesthetized animals were decapitated and the left cochleae harvested and prepared for hair cell quantification. The temporal bones were removed and placed in 4% paraformaldehyde in phosphate buffer. The bone from the apical tip of the cochleae was removed and the round and oval windows were opened under stereoscopic magnification to allow for the flow of fixative. Cochleae were decalcified in a 5% EDTA solution for 24 hours prior to processing. The otic capsule was then trimmed to reveal the organ of Corti. The organ of Corti was separated in several fragments from the modiolus and the lateral wall tissues. Triton X-100 (0.3%, 15 min.) was used to permeabilize the samples, then the samples were incubated for 60 min. in 1:100 Alexa Fluor 594 phalloidin and PBS. The samples were rinsed and mounted using Fluoro-Gel with Tris Buffer (Electron Microscopy Sciences, Hatfield, PA) on glass slides. Tissues were then analyzed using an epifluorescence microscope (Leica DMRB) using a standard TRITC filter and ×10, ×40, ×50 and ×100 oil objectives. Diap3-Tg animal cochleae were only analyzed qualitatively. In *Gjb2*-CKO mice cochleae hair cells were quantified. A 0.19 mm calibrated scale was imposed on the right objective field. The rows of hair cells were oriented longitudinally along the scale. Beginning at the apex, each 0.19 mm section was analyzed for the presence or absence of hair cells. Manual counts were analyzed using our in-house Excel based program (KHRI Cytocochleogram, version 3.0.6) and compared to a data base of normal CBA/J mouse cochleae.

### Statistical analysis

Multivariate analysis of variance was performed on thresholds at specified time points and hair cell counts at termination. To account for differences in initial thresholds for tests of changes over time, we analyzed the difference between initial and ending thresholds (threshold shifts). Post hoc tests evaluating the contribution of individual variables were judged using sequential Bonferroni criterion to maintain table-wide alpha = 0.05. In multiple group analyses at a single time point, post-hoc two group comparisons were evaluated using Tukey’s HSD test. All analyses were performed in R (Version 3.2.2, http://cran.r-project.org/).

## Additional Information

**How to cite this article**: Green, K. L. *et al.* ACEMg Diet Supplement Modifies Progression of Hereditary Deafness. *Sci. Rep.* **6**, 22690; doi: 10.1038/srep22690 (2016).

## Figures and Tables

**Table 1:** Sample sizes for diet intervention groups for *Diap3*-Tg and *Gjb2*-CKO mice.

*Diap3*-Tg		*Gjb2*-CKO			
ACEMg	Control	*Post-Weaning*		*Prenatal*	
6	5	**ACEMg**	**Control**	**ACEMg**	**Control**
		6	8	17	13
