# Corrigendum: ACEMg Diet Supplement Modifies Progression of Hereditary Deafness

**DOI:** 10.1038/srep24469

**Published:** 2016-04-20

**Authors:** Kari L. Green, Donald L. Swiderski, Diane M. Prieskorn, Susan J. DeRemer, Lisa A. Beyer, Josef M. Miller, Glenn E. Green, Yehoash Raphael

Scientific Reports 6: Article number: 2269010.1038/srep22690; published online: 03112016; updated: 04202016

This Article contains errors in the legend of Fig. 5.

“Post-weaning dietary supplement slowed or reversed hearing loss in *Diap3*-Tg mutant mice.”

should read:

“Post-weaning dietary supplement accelerated hearing loss in *Diap3*-Tg mutant mice.”

